# Association of early menarche with breast tumor molecular features and recurrence

**DOI:** 10.1186/s13058-024-01839-0

**Published:** 2024-06-17

**Authors:** Alexandra R. Harris, Tengteng Wang, Yujing J. Heng, Gabrielle M. Baker, Phuong Anh Le, Jun Wang, Christine Ambrosone, Adam Brufsky, Fergus J. Couch, Francesmary Modugno, Christopher G. Scott, Celine M. Vachon, Susan E. Hankinson, Bernard A. Rosner, Rulla M. Tamimi, Cheng Peng, A. Heather Eliassen

**Affiliations:** 1 Channing Division of Network Medicine, Brigham and Women’s Hospital, Boston, MA USA; 2 Department of Epidemiology, Harvard T.H. Chan School of Public Health, Boston, MA USA; 3 Integrative Tumor Epidemiology Branch, Division of Cancer Epidemiology and Genetics, National Cancer Institute, National Institutes of Health, 9609 Medical Center Drive, Bethesda, MD 20892 USA; 4 Department of Pathology, Harvard Medical School, Boston, MA USA; 5 Department of Pathology, Beth Israel Deaconess Medical Center, Harvard Medical School, Boston, MA USA; 6 Department of Population and Public Health Sciences, Keck School of Medicine, University of Southern California, Los Angeles, CA USA; 7 Department of Cancer Prevention and Control, Roswell Park Comprehensive Cancer Center, Buffalo, NY USA; 8 Department of Medicine, University of Pittsburgh Medical Center, Pittsburgh, PN USA; 9 Department of Laboratory Medicine and Pathology, Mayo Clinic, Rochester, MN USA; 10 Division of Gynecologic Oncology, Department of Obstetrics, Gynecology and Reproductive Sciences, University of Pittsburgh School of Medicine, Pittsburgh, PA USA; 11 Department of Epidemiology, School of Public Health, University of Pittsburgh, Pittsburgh, PA USA; 12 Women’s Cancer Research Center, Magee-Womens Research Institute and Hillman Cancer Center, Pittsburgh, PA USA; 13 Department of Quantitative Health Sciences, Mayo Clinic, Rochester, MN USA; 14 Department of Biostatistics and Epidemiology, School of Public Health and Health Sciences, University of Massachusetts Amherst, Amherst, MA USA; 15 Department of Biostatistics, Harvard T.H. Chan School of Public Health, Boston, MA USA; 16 Department of Population Health Sciences, Weill Cornell Medical College, New York, NY USA

**Keywords:** Age at menarche, Reproductive risk factors of breast cancer, Early-life risk factors of breast tumor biology, Breast cancer recurrence, Early menarche gene expression signature

## Abstract

**Background:**

Early menarche is an established risk factor for breast cancer but its molecular contribution to tumor biology and prognosis remains unclear.

**Methods:**

We profiled transcriptome-wide gene expression in breast tumors (N = 846) and tumor-adjacent normal tissues (N = 666) from women in the Nurses’ Health Studies (NHS) to investigate whether early menarche (age < 12) is associated with tumor molecular and prognostic features in women with breast cancer. Multivariable linear regression and pathway analyses using competitive gene set enrichment analysis were conducted in both tumor and adjacent-normal tissue and externally validated in TCGA (N = 116). Subgroup analyses stratified on ER-status based on the tumor were also performed. PAM50 signatures were used for tumor molecular subtyping and to generate proliferation and risk of recurrence scores. We created a gene expression score using LASSO regression to capture early menarche based on 28 genes from FDR-significant pathways in breast tumor tissue in NHS and tested its association with 10-year disease-free survival in both NHS (N = 836) and METABRIC (N = 952).

**Results:**

Early menarche was significantly associated with 369 individual genes in adjacent-normal tissues implicated in extracellular matrix, cell adhesion, and invasion (FDR ≤ 0.1). Early menarche was associated with upregulation of cancer hallmark pathways (18 significant pathways in tumor, 23 in tumor-adjacent normal, FDR ≤ 0.1) related to proliferation (e.g. Myc, PI3K/AKT/mTOR, cell cycle), oxidative stress (e.g. oxidative phosphorylation, unfolded protein response), and inflammation (e.g. pro-inflammatory cytokines IFN$\alpha$ and IFN$\gamma$). Replication in TCGA confirmed these trends. Early menarche was associated with significantly higher PAM50 proliferation scores (β = 0.082 [0.02–0.14]), odds of aggressive molecular tumor subtypes (basal-like, OR = 1.84 [1.18–2.85] and HER2-enriched, OR = 2.32 [1.46–3.69]), and PAM50 risk of recurrence score (β = 4.81 [1.71–7.92]). Our NHS-derived early menarche gene expression signature was significantly associated with worse 10-year disease-free survival in METABRIC (N = 952, HR = 1.58 [1.10–2.25]).

**Conclusions:**

Early menarche is associated with more aggressive molecular tumor characteristics and its gene expression signature within tumors is associated with worse 10-year disease-free survival among women with breast cancer. As the age of onset of menarche continues to decline, understanding its relationship to breast tumor characteristics and prognosis may lead to novel secondary prevention strategies.

**Supplementary Information:**

The online version contains supplementary material available at 10.1186/s13058-024-01839-0.

## Introduction

Early menarche, the onset of the menstrual cycle at an early age (< 12 years old, the median age at menarche in the United States), is consistently associated with increased breast cancer risk [1, 2]. In large, pooled studies and meta-analyses, each year of younger onset of menarche was associated with a 5–9% increased risk of breast cancer [1–3]. The increase in breast cancer risk due to lengthening of reproductive cycling is thought to arise from higher levels and longer exposure time to estrogens [1, 2], which are known mitogens and drive a number of cancers [4]. However, no prior study to our knowledge has comprehensively investigated tumor molecular characteristics associated with early menarche. Further, the impact of early menarche on breast cancer prognosis also remains largely under-studied. Data from the National Health and Nutrition Examination Survey (NHANES) reveal that average ages of menarche declined by as much as 11 months between 1920 and 1980, with non-Hispanic Black women showing the largest decline in mean age of menarche (14 months) [5]. As the decreasing secular trend of age of onset of menarche continues [6], understanding the underlying mechanisms through which early menarche is associated with tumorigenesis could lead to novel prevention strategies. However, there is a general paucity of data linking early life exposures, such as age of menarche, with molecular tumor features that occur decades later in life. Here, we utilized the long-term prospective epidemiological data and enriched tumor molecular data in the Nurses’ Health Studies (NHS), as well as validation using The Cancer Genome Atlas (TCGA) and Molecular Taxonomy of Breast Cancer International Consortium (METABRIC) databases, to investigate how age at menarche may be associated with tumor molecular characteristics and prognosis in women with breast cancer.

## Methods

### Study population

We used data from two large-scale prospective cohorts of registered female nurses in the United States, the NHS and NHSII. NHS (established in 1976) recruited 121,701 women between ages 30 and 55 years and NHSII (initiated in 1989) enrolled 116,429 women between ages 25 and 42 years. In both cohorts, participants completed an initial study questionnaire and subsequent questionnaires biennially afterward; cumulative follow-up rates were greater than 90% [7]. As described previously [8], invasive breast cancer cases were identified initially by questionnaire (start of follow-up to 2012) or National Death Index search upon lack of participant response; breast cancer cases were confirmed by centralized medical record review using established protocols. No breast cancer cases included in our analysis had any prior personal history of cancer. Completion of the questionnaire was considered to imply informed consent upon study protocol approval by the Institutional Review Boards of the Brigham and Women's Hospital (Boston, MA) and Harvard T.H. Chan School of Public Health (Boston, MA) in 1976 (NHS) and 1989 (NHSII). NHSI/II were conducted in accordance with recognized ethical guidelines (Declaration of Helsinki).

### Gene expression measurements

954 incident breast cancer cases within the study were eligible for transcriptomic analysis [10], which had participant characteristics similar to those without gene expression data. RNA was extracted from multiple cores of 1 or 1.5 mm procured from FFPE tumor (n = 1–3 cores) and matched normal adjacent tissue taken from > 1 cm from the tumor margin during surgery (n = 3–5 cores) and isolated using the Qiagen AllPrep RNA Isolation Kit. Transcriptome-wide gene expression was profiled using Affymetrix Glue Grant Human Transcriptome Array 3.0 (hGlue 3.0) and Human Transcriptome Array 2.0 (HTA 2.0) microarray chips. Normalization was performed using robust multiarray averages. Data was log-2-transformed and Affymetrix Power Tools probeset summarization-based metrics were used for quality control. After quality control and exclusion based on missing data, 846 tumor and 666 normal-adjacent tissues were used in this analysis. Gene expression data was deposited in Gene Expression Omnibus and is publicly available (GEO#; GPL22920, GSE93601, GSE115577). Of note, participant characteristics of breast cancer cases with and without gene expression were similar [11]. The most variable probe was selected to represent the gene when genes were mapped by multiple probes. *ComBat,* which is an empirical Bayes method for batch effects, was used to control for technical variabilities [12]. Genes with expression in the lowest quartile (< 25%) were excluded from the analyses.

### Exposure and covariates

Age at menarche (age in years) and height (measured in feet and inches) were reported on the baseline study questionnaire. Weight at age 18 was reported during 1980 questionnaire cycle for NHSI and baseline study questionnaire for NHSII. Race was reported during 1992 questionnaire cycle for NHSI and baseline study questionnaire for NHSII. History of oral contraceptive use, menopausal status, parity, age at breast cancer diagnosis, calendar year of breast cancer diagnosis, weight and physical activity level at time of diagnosis were obtained via the biennial NHS and NHSII questionnaires. BMI was calculated by dividing the participant’s weight in kilograms by their height in meters squared (kg/m^2^). Tumor characteristics (stage and grade), treatment information (chemotherapy, radiotherapy, and endocrine therapy) were obtained from medical records or supplemental questionnaire. Estrogen receptor (ER) status was determined after central review of breast cancer tissue microarrays (TMAs), or pathology reports if missing. Based on previous literature, we defined early menarche as menstruation occurring before age 12, the median age at menarche in the U.S [9]. Therefore, age at menarche, our primary exposure, was dichotomized and modeled as a categorical variable of “early” (< 12 years old) vs. “later” (≥ 12 years old). Our analyses were restricted to complete cases that included the following covariates, selected a priori: age at breast cancer diagnosis (continuous), year of diagnosis (continuous), tumor stage (1–4), chemotherapy (yes/no/unknown), radiation (yes/no/unknown), endocrine therapy (yes/no/unknown), oral contraceptive use (current user/past user/never user/unknown), race (White/non-White), parity (continuous), BMI at age 18 (continuous), BMI change (BMI at diagnosis – BMI at age 18), and physical activity at time of diagnosis (continuous). Within our analytic cohort with gene expression measurements, 52 cases were excluded from analysis due to missing information on age at menarche; missing BMI and physical activity data were imputed using the median. Secondary analyses were also performed modeling age at menarche as continuous.

### Breast cancer recurrence

Breast cancer recurrences were determined as previously described [13]. Briefly, supplemental questionnaires were sent to living cohort members with a previously confirmed diagnosis of breast cancer. Reports of new cancers of the liver, bone, brain, and lung—common sites of breast cancer metastasis—following their breast cancer diagnosis were considered breast cancer recurrences. Participants who died from breast cancer without previous report of recurrence were also presumed to have a breast cancer recurrence. The time scale of disease-free survival is thus defined as the time from initial diagnosis to either recurrence or end of follow-up, with participants who died of other causes censored at time of death. Deaths were most commonly reported by families, and deaths among nonrespondents were identified through the National Death Index, as is consistent in previous NHS analyses [14]. Once a death was reported, the specific cause was subsequently determined by medical record review or death certificate.

### Statistical analysis

#### Age at menarche and gene expression

We evaluated the association between age at menarche with transcriptome-wide gene expression for each individual gene using covariate-adjusted linear regression (*limma*) [15]. Each regression model was adjusted for confounders determined a priori and surrogate variables generated from the transcriptome data (the leek method from Bioconductor *sva* package in R) [16]. Analyses were performed on tumor and tumor-adjacent tissues separately. Subgroup analyses stratified on ER-status based on the tumor. We used an FDR ≤ 0.1 to determine whether a gene meets transcriptome-wide significance [17]. Functional enrichment of biological pathways associated with age of menarche was performed using Correlation Adjusted Mean Rank (CAMERA), a competitive gene set test [18] using an intergene correlation of 0.01. The same set of covariates used in the single gene analysis are controlled for here. We used the 50 cancer “hallmark” gene sets from the Molecular Signature Database (MSigDB; http://www.broadinstitute.org/gsea/msigdb/) in our pathway analyses and an FDR ≤ 0.1 to determine statistical significance. For external validation, pathway analyses were replicated in a small subset of TCGA for which RNA-sequencing data and information on age at menarche was available (N = 116) [10, 19]. For this validation dataset, six TCGA sites were originally contacted and data from three (Roswell Park Cancer Institute, University of Pittsburgh, Mayo Clinic) sites that agreed to collect or provide breast cancer risk factor data on these cases were included in this study, as previously described [20]. Covariates from TCGA were selected a priori to match those used in the NHS analysis as closely as possible, though there are some differences: age at breast cancer diagnosis (continuous), year of diagnosis (continuous), tumor stage (1–4), race (White/non-White), parity (continuous), BMI at diagnosis (continuous), ER status (yes/no), and menopausal status (yes/no). Pathway analyses were again performed using CAMERA as described above and hits with FDR ≤ 0.2 were considered significant for validation.

#### Age at menarche and PAM50 scores

Proliferation score and risk of recurrence scores based on PAM50 assay were computed as described previously in NHS [21]. Briefly, proliferation score is computed by averaging gene expression of 11 genes (*BIRC5*, *CCNB1*, *CDC20*, *NUF2*, *CEP55*, *NDC80*, *MKI67*, *PTTG1*, *RRM2*, *TYMS* and *UBE2C*) [22]. Risk of recurrence (ROR) score combines gene expression of 50 gene in the PAM50 assay with tumor size and nodal status to compute an integer score proportional to risk of recurrence (0–100). PAM50 ROR score has been found to be highly predictive of risk of distant relapse [23]. Multiple linear regression was performed using these scores as continuous dependent variables and age at menarche as the main predictor. PAM50 is frequently used to classify breast tumors based on their gene expression into five molecular subtypes that differ both in biological characteristics and prognosis [24]. Multinomial regression was performed to test association of early menarche with PAM50-based intrinsic molecular subtypes (luminal B, normal, HER2, and basal) compared to the least aggressive luminal A subtype [24]. All other covariates previously mentioned were used for adjustment.

#### Early menarche-derived gene expression signature and breast cancer recurrence

To examine associations between an early menarche-associated gene expression signature and breast cancer recurrence, we included individual genes from FDR-significant pathways in the tumor (receptor-agnostic) showing nominal significance (p ≤ 0.05) to create a gene expression score, calculated as ∑(z-transformed genes from positively regulated pathways)—∑(z-transformed genes from negatively regulated pathways). LASSO regression was used in *glmnet* in R to select the most predictive genes while preventing overfitting through shrinkage of the regression coefficients [25].

*Discovery cohort: Nurses’ Health Studies* We used Cox proportional hazards regression to examine the association between our menarche-associated gene expression signature and breast cancer recurrence among stage 1–3 breast cancer cases. Scores were modeled as categorical, using quartiles of expression as cut points to make scores from 1 to 4, with 1 representing the lowest score (most dissimilar to early menarche) and 4 representing the highest score (most similar to early menarche). Hazard ratios and 95% confidence intervals were calculated. Recurrence-free survival was defined as the time between cancer diagnosis and either recurrence (cancer detected at common metastatic sites, such as bone, brain, lungs, and liver) or death from breast cancer without reported recurrence (12). We evaluated an interaction term between score and the log of recurrence time to test violation of the proportional hazards assumption with a likelihood ratio test. Proportional hazards were violated; we therefore applied a piecewise Cox model using the crossing of the Kaplan Meier curves as our cut point, which was 10 years.

*Validation cohort: METABRIC* To assess the generalizability of our menarche-associated gene expression signature, we leveraged an independent dataset, the Molecular Taxonomy of Breast Cancer International Consortium (METABRIC), for validation. Using our NHS-derived gene signature, we computed the early menarche-associated score in tumors from METABRIC using the available gene expression data. We then used a Cox regression model to assess the association of the score with breast cancer recurrence. Covariates included: age at diagnosis, ER status, batch, menopausal status, cancer stage, and treatment (chemotherapy, radiotherapy, and/or hormonal therapy). Analysis was restricted to complete cases to include a total of 952 breast cancer cases, stage 1–3 only. Score was again modeled as categorical, using quartiles of expression in METABRIC as cut points. We evaluated an interaction term between score and the log of recurrence time to test violation of the proportional hazards assumption; no violation was found.

## Results

### Participant characteristics

Of the 846 NHS women with tumor gene expression data, 206 had early menarche (< 12 years of age) (Table 1). Demographics were similar between women with early or later menarche, including age and year at breast cancer diagnosis and race, which is predominantly White (~ 95%) in the NHS cohort. Nearly 70% of women were postmenopausal at diagnosis in both the early and later menarche groups. Frequencies of breast cancer stage were similar between groups, consisting of mostly stage 1–3 tumors. Modest differences were observed in therapies used in disease management, with radiation therapy more commonly used among women with later age at menarche than early menarche (52.8% vs. 33.5%, respectively). Conversely, 51.0% of those with early menarche received chemotherapy compared to 43.3% of those with later menarche. Endocrine therapy and oral contraceptive use were similar between groups. Women in both groups had a median of two children and similar levels of physical activity.

**Table 1:** Participant characteristics of breast cancer cases in the NHS with tissue gene expression data (N = 846)

Covariate	Age at menarche	
	Early	Later
	(< 12 years old)	(≥ 12 years old)
	N = 206	N = 640
Age at breast cancer diagnosis [mean (SD)]	58.2 (10.4)	59.9 (11.5)
Year of breast cancer diagnosis [median (IQR)]	1999 (8)	1999 (8)
Breast cancer stage [n (%)]		
1	122 (59%)	387 (60%)
2	67 (33%)	192 (30%)
3	15 (7%)	57 (9%)
4	2 (1%)	4 (1%)
Estrogen receptor status [n (%)]		
Positive	166 (81%)	518 (90%)
Negative	40 (19%)	122 (19%)
Chemotherapy [n (%)]	105 (51%)	277 (43%)
Radiation therapy [n (%)]	69 (34%)	338 (53%)
Endocrine therapy [n (%)]	138 (67%)	411 (64%)
Oral contraceptive use at diagnosis [n (%)]		
Current user	9 (4%)	36 (6%)
Past user	125 (61%)	379 (59%)
Never user	71 (35%)	224 (35%)
Race [n (%)]		
White	197 (96%)	610 (95%)
Non-White	9 (4%)	30 (5%)
Parity [median (IQR)]	2 (2)	2 (1)
BMI at 18 years old, kg/m^2^ [mean (SD)]	21.5 (5.7)	20.7 (4.9)
Physical activity at diagnosis, MET^a^-hrs/wk [median (IQR)]	11.0 (23.9)	12.1 (21.8)
Postmenopausal at diagnosis [n (%)]	141 (68%)	450 (70%)

^a^MET: Metabolic equivalent task

### Gene expression and pathway analysis in women who experienced early menarche

**Fig. 1:**
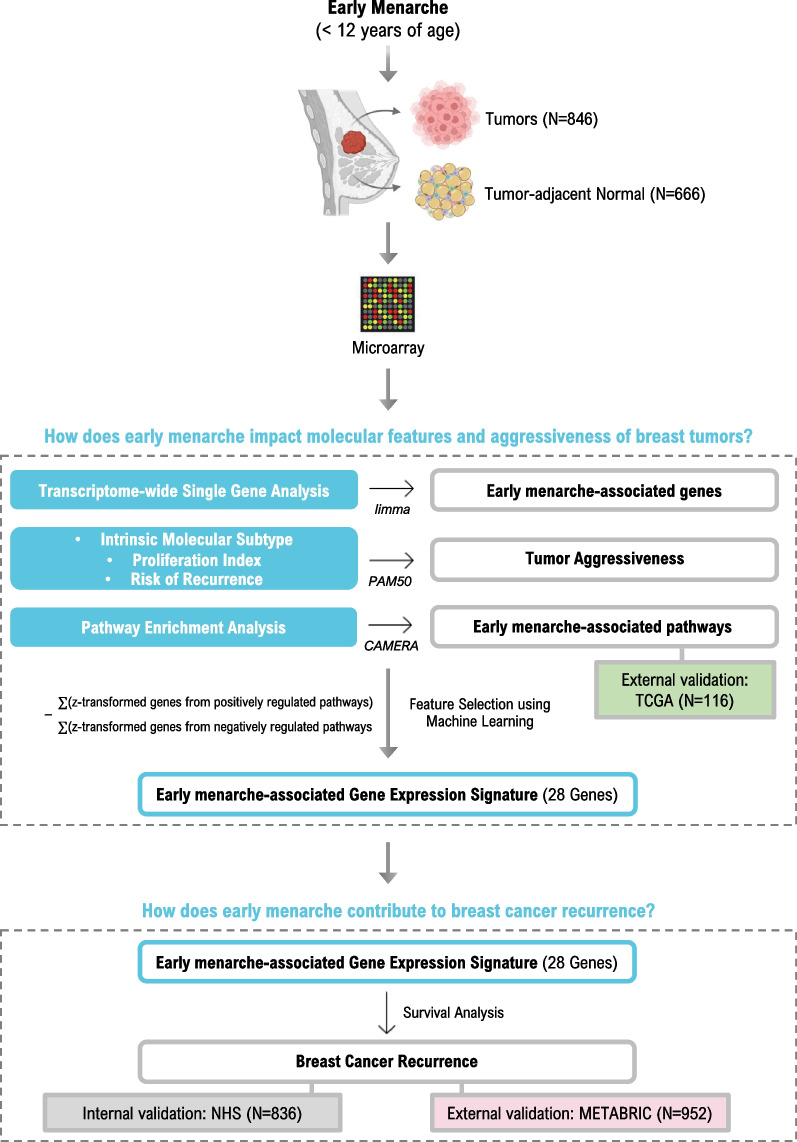
Study workflow of gene expression analyses

A full study workflow is presented in Fig. 1. We did not observe a statistically significant association between early menarche and any individual gene in tumor tissues. However, upon stratification by ER status, we identified 28 genes associated with early menarche in ER-positive tumors (FDR ≤ 0.1) (Supplemental Fig. 1, Table S1). Among these were genes associated with Notch and TGFß signaling pathways (*DMXL2* and *LRRC32*, respectively), DNA damage (*HUS1*), cell stress and metastatic cell survival (*ERLEC1*), extracellular matrix (*COL16A1*), and proliferation (*PIK3C3*). However, no single genes were significant in ER-negative tumors. In the matched tumor-adjacent normal tissue, 369 individual genes were significantly associated with early menarche (FDR ≤ 0.1) (Table S2). Included among these were genes positively associated with extracellular matrix, cell adhesion, and invasion (e.g., RHOA, RAB1A, CTNND1, ITFG1, CAV1, CST3), cell cycle/proliferation (e.g., CDC16, CDC42, GSK3B, MAPKA5), and other genes with known roles in tissue transformation. Further stratification by estrogen receptor status found early menarche was significantly associated with 9 genes in normal tissues adjacent to ER+ tumors and 0 in normal tissues adjacent to ER− tumors. When comparing the 28 significant genes in ER+ tumor tissues and the 9 significant genes in ER+ adjacent normal tissues, 2 genes overlapped (HUS1 and PRKAG3).

In multivariable-adjusted competitive gene set enrichment analysis, 18 cancer hallmark pathways were significantly associated (FDR ≤ 0.1) with early menarche in tumor tissues; 23 pathways were significantly associated with early menarche in tumor-adjacent normal tissues (Table 2). Fifteen enriched pathways overlapped between tumor and normal tissues. In both tissue types, early menarche was associated with upregulation of pathways associated with proliferation (e.g. Myc, PI3K/AKT/mTOR, cell cycle), oxidative stress (e.g. oxidative phosphorylation, unfolded protein response), and inflammation (e.g. pro-inflammatory cytokines IFN$\alpha$ and IFN$\gamma$). Further, in both tissues, early menarche was associated with upregulation of adipogenesis. Normal tissues also showed enrichment of unique pathways like epithelial to mesenchymal transition (EMT) and angiogenesis, features indicative of a cancer-promoting microenvironment and tissue transformation, and downregulation of myogenesis.

**Table 2:** Pathway enrichment analysis of age at menarche in breast tumors and normal-adjacent tissues

	*N* genes	Direction	*P* value	FDR
*Tumor (N* = *846)*				
Proliferation and mitogenic effects				
HALLMARK_MYC_TARGETS_V1	187	Up	1.30E−15	6.48E−14
HALLMARK_KRAS_SIGNALING_DN	140	Down	1.58E−07	1.97E−06
HALLMARK_MTORC1_SIGNALING	165	Up	3.49E−06	2.91E−05
HALLMARK_G2M_CHECKPOINT	149	Up	1.45E−05	9.94E−05
HALLMARK_E2F_TARGETS	147	Up	0.0002	0.0010
HALLMARK_PI3K_AKT_MTOR_SIGNALING	92	Up	0.0010	0.0040
HALLMARK_ANDROGEN_RESPONSE	88	Up	0.0015	0.0058
HALLMARK_MITOTIC_SPINDLE	166	Up	0.0102	0.0319
HALLMARK_HEDGEHOG_SIGNALING	31	Down	0.0210	0.0619
HALLMARK_TGF_BETA_SIGNALING	53	Up	0.0268	0.0744
Cellular stress				
HALLMARK_PROTEIN_SECRETION	83	Up	2.46E−10	6.14E-09
HALLMARK_OXIDATIVE_PHOSPHORYLATION	185	Up	1.07E−09	1.78E-08
HALLMARK_UNFOLDED_PROTEIN_RESPONSE	105	Up	9.93E−07	9.93E-06
HALLMARK_DNA_REPAIR	129	Up	0.009	0.030
Inflammation				
HALLMARK_INTERFERON_ALPHA_RESPONSE	76	Up	1.59E−05	9.94E-05
HALLMARK_INTERFERON_GAMMA_RESPONSE	154	Up	2.17E−05	0.0001
Tumor Microenvironment				
HALLMARK_MYOGENESIS	178	Down	0.0002	0.0010
HALLMARK_ADIPOGENESIS	183	Up	0.0064	0.0229
*Tumor-Adjacent Normal (N = 666)*				
Proliferation and mitogenic effects				
HALLMARK_MYC_TARGETS_V1	187	Up	1.06E−18	5.30E−17
HALLMARK_KRAS_SIGNALING_DN	140	Down	3.48E−06	3.48E−05
HALLMARK_ANDROGEN_RESPONSE	88	Up	1.19E−05	8.53E−05
HALLMARK_MTORC1_SIGNALING	165	Up	1.49E−05	9.31E−05
HALLMARK_MITOTIC_SPINDLE	166	Up	0.0000	0.0002
HALLMARK_G2M_CHECKPOINT	149	Up	0.0001	0.0003
HALLMARK_TGF_BETA_SIGNALING	53	Up	0.0002	0.0009
HALLMARK_PI3K_AKT_MTOR_SIGNALING	92	Up	0.0003	0.0010
HALLMARK_CHOLESTEROL_HOMEOSTASIS	64	Up	0.0151	0.0379
HALLMARK_E2F_TARGETS	147	Up	0.0191	0.0454
Cellular stress				
HALLMARK_PROTEIN_SECRETION	83	Up	2.31E−13	5.79E−12
HALLMARK_OXIDATIVE_PHOSPHORYLATION	185	Up	7.68E−13	1.28E−11
HALLMARK_UNFOLDED_PROTEIN_RESPONSE	105	Up	7.95E−07	9.94E−06
HALLMARK_APOPTOSIS	142	Up	6.79E−05	0.0003
HALLMARK_UV_RESPONSE_DN	128	Up	6.83E−05	0.0003
HALLMARK_REACTIVE_OXIGEN_SPECIES_PATHWAY	42	Up	0.015	0.0379
Inflammation				
HALLMARK_INTERFERON_ALPHA_RESPONSE	76	Up	0.0047	0.0148
HALLMARK_INTERFERON_GAMMA_RESPONSE	154	Up	1.14E−02	0.0316
Cancer metabolism				
HALLMARK_FATTY_ACID_METABOLISM	124	Up	3.27E−03	0.0109
HALLMARK_HEME_METABOLISM	173	Up	2.08E−02	0.0472
Tumor microenvironment				
HALLMARK_ADIPOGENESIS	183	Up	0.0000	0.0000
HALLMARK_EPITHELIAL_MESENCHYMAL_TRANSITION	174	Up	0.0056	0.0165
HALLMARK_ANGIOGENESIS	30	Up	0.0403	0.0877

*Note* Each regression model was adjusted for the following covariates, selected a priori: age at breast cancer diagnosis (continuous), year of diagnosis (continuous), tumor stage (1-4), chemotherapy (yes/no/unknown), radiation (yes/no/unknown), endocrine therapy (yes/no/unknown), oral contraceptive use (current-/past-/never-user/unknown), race (White/non-White), parity (continuous), BMI at 18 (continuous), weight change (BMI at diagnosis – BMI at 18), and physical activity at time of diagnosis (continuous). Age at menarche was dichotomized and modeled as a categorical variable of “early” (< 12 years old) vs. “later” (> 12 years old)

Stratified by estrogen receptor status, 19 significant pathways were observed in ER-positive tumor tissue and 21 in matched ER-positive normal tissue, all of which mirrored the unstratified analysis with upregulation of pathways involved in proliferation, cell stress, and cancer cell metabolism (Table S3); similar findings were observed in ER-negative tissues, with 9 pathways in tumor and 22 pathways in tumor-adjacent normal tissues detected with an FDR ≤ 0.1 (Table S4). Analyses modeling age at menarche as continuous had very similar results (Table S5-S7), where we observed that increasing age at menarche was associated with downregulation of proliferation and cellular stress pathways.

We replicated the pathway analysis in TCGA (Table 3, Table S8). Although limited by sample size (N = 116), early menarche was associated with an upregulation of many of the same proliferation-related signaling pathways (MTORC1 signaling: FDR = 0.005; PI3K-AKT-MTOR signaling: FDR = 0.05), cellular stress pathways (Protein Secretion: FDR = 0.02), and inflammation pathways (Interferon Gamma Response: FDR = 0.11) that we observed in NHS. In addition, we observed the upregulation of several biologically related but unique pathways associated with early menarche, such as those involved in estrogen response (Estrogen Response Early: FDR = 0.05; Estrogen Response Late: FDR = 0.06) and others involved in inflammation and innate immune response (Allograft Rejection: FDR = 0.05; Complement: FDR = 0.06; Coagulation: FDR = 0.12).

**Table 3:** Pathway enrichment analysis of age at menarche in TCGA

	***N* genes**	**Direction**	**P value**	**FDR**
*Tumor (N = 156)*				
Proliferation and mitogenic effects				
HALLMARK_MTORC1_SIGNALING	195	Up	2.48e−06	6.19e−05
HALLMARK_E2F_TARGETS	195	Up	9.73e−04	1.62e−02
HALLMARK_G2M_CHECKPOINT	188	Up	3.45e−03	3.45e−02
HALLMARK_PI3K_AKT_MTOR_SIGNALING	99	Up	1.29e−02	6.47e−02
Cellular stress				
HALLMARK_GLYCOLYSIS	184	Up	1.52e−03	1.91e−02
HALLMARK_PROTEIN_SECRETION	93	Up	4.48e−03	3.73e−02
Hormonal Signaling				
HALLMARK_ESTROGEN_RESPONSE_LATE	188	Up	1.28e−02	6.47e−02
Inflammation				
HALLMARK_COAGULATION	107	Up	9.12e−03	5.70e−02
Tumor microenvironment				
HALLMARK_EPITHELIAL_MESENCHYMAL_TRANSITION	193	Up	1.88e-06	6.19e−05
HALLMARK_APICAL_JUNCTION	173	Up	6.73e−03	4.81e−02

*Note* This regression model was adjusted for the following covariates, selected a priori: age at breast cancer diagnosis (continuous), year of diagnosis (continuous), tumor stage (1-4), tumor grade (1-4), race (White/non-White), parity (continuous), BMI at diagnosis (continuous), estrogen receptor status (yes/no), and menopausal status (yes/no). Significant hits with FDR<0.2 were considered for validation. Age at menarche was dichotomized and modeled as a categorical variable of “early” (< 12 years old) vs. “later” (> 12 years old)

In PAM50 analyses (Table 4) of molecular subtype, early menarche was associated with increased odds of HER2-enriched (OR = 2.32 [1.46–3.69]) and basal-like (OR = 1.84 [1.18–2.85]) breast cancer subtypes relative to luminal A. Early menarche was associated with a significant increase in PAM50 proliferation score ($\beta$=0.082 [0.02–0.14], p = 0.009) and a higher risk of breast cancer recurrence, with an estimated increase of 4.81 in the PAM50 ROR score ($\beta$=4.81 [1.71–7.92], p = 0.003).

**Table 4:** Early menarche is associated with more proliferative and aggressive tumors in breast cancer

	Early Menarche^c^	
Intrinsic molecular tumor subtype^a^	OR (95% CI)	*P* value
Luminal A	reference	reference
Normal	1.12 (0.73, 1.71)	0.6126
Luminal B	1.74 (1.08, 2.80)	0.0235
HER2-enriched	2.32 (1.46, 3.69)	0.0004
Basal	1.84 (1.18, 2.85)	0.0068

Tumor proliferation^b^	β (95% CI)	*P* value
PAM50 proliferation index	0.082 (0.021, 0.142)	0.0085

Disease recurrence^b^	β (95% CI)	*P* value
PAM50 risk of recurrence score	4.81 (1.71, 7.92)	0.0025

^a^Multinomial regression model with categorical dependent variables (PAM50 intrinsic molecular subtypes)

^b^Linear regression model with continuous dependent variables (PAM50 proliferation and ROR scores)

^c^Covariates: age at breast cancer diagnosis (continuous), year of diagnosis (continuous), tumor stage (1–4), chemotherapy (yes/no/unknown), radiation (yes/no/unknown), endocrine therapy (yes/no/unknown), oral contraceptive use (current user/past user/never user/unknown), race (White/non-White), parity (continuous), BMI at 18 years of age (continuous), weight change (BMI at diagnosis – BMI at 18 years of age), and physical activity at time of diagnosis (continuous)

Age at menarche was dichotomized and modeled as a categorical variable of “early” (< 12 years old) versus “normal” (≥ 12 years old)

Score Range: PAM50 Proliferation Score Range: −1 to 1, PAM50 Risk of Recurrence Score Range: 0–100

### Early menarche-derived gene expression signature and risk of breast cancer recurrence in NHS and METABRIC

**Fig. 2:**
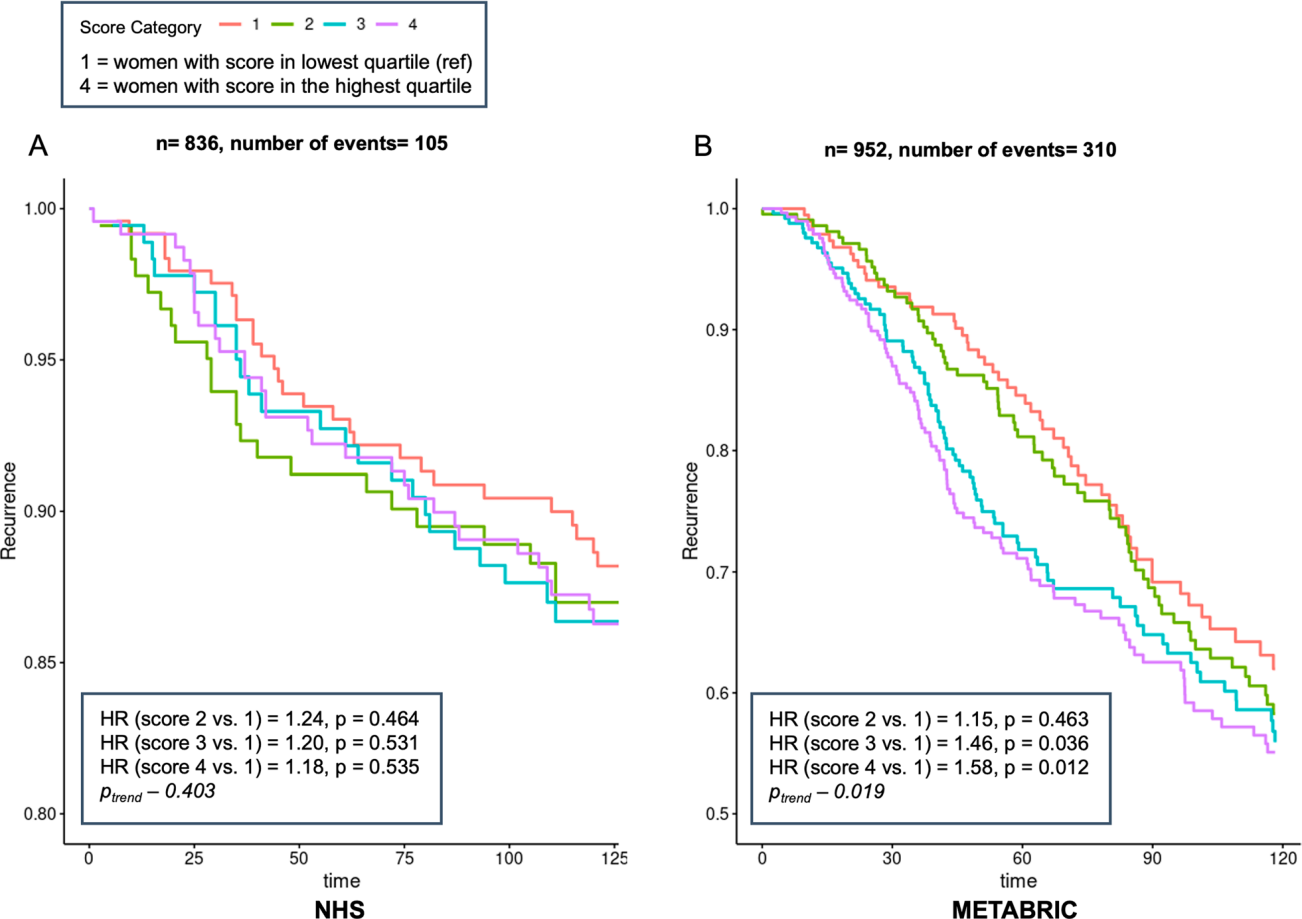
Association between early menarche and 10-year disease-free survival. Covariate−adjusted marginal survivor curves computed from Cox proportional hazards models for 10−year disease−free survival and early menarche gene expression score modelled as quartiles of expression, with 1 representing the lowest level of expression (the most dissimilar to early menarche) and 4 representing the highest level of expression (most representative of early menarche signature), in NHS ( **A** ) and METABRIC ( **B **) cohorts. Covariates in NHS included age at breast cancer diagnosis (continuous), year of diagnosis (continuous), estrogen receptor status (yes/no), tumor stage (1–3), tumor grade (1–4), chemotherapy (yes/no/unknown), radiation (yes/no/unknown), endocrine therapy (yes/no/unknown), oral contraceptive use (current user/past user/never user/unknown), race (White/non-White), parity (continuous), BMI at 18 years of age (continuous), weight change (BMI at diagnosis – BMI at 18 years of age), and physical activity at time of diagnosis (continuous). Covariates in METABRIC attempted to mirror NHS covariates as closely as possible, though not all were available; they included age at diagnosis, estrogen receptor status, batch, menopausal status, cancer stage, and treatment (chemotherapy, radiotherapy, and/or hormonal therapy)

We next created an early menarche signature based on 28 genes selected from LASSO regularization regression and examined its association with 10-year disease-free survival (Table 5). The majority of the 28 genes included within the signature were involved in three main biological processes: (1) cell stress response and metabolism (e.g. BAG1, HUS1, DRAM2); (2) cell proliferation, differentiation, and invasion (e.g. and (3) inflammation. In the NHS, individuals with higher early menarche-associated gene expression scores had an 18% increased risk of recurrence, though not statistically significant (N = 836, N_events_ = 105, HR of highest vs. lowest score quartile = 1.18 [0.70–2.0], p-*trend* = 0.403, Fig. 2A). In METABRIC, after covariate adjustment, higher early menarche-associated gene expression score (based on the same 28 genes identified originally in NHS) was associated with a 58% higher risk of cancer recurrence (N = 952, N_events_ = 310, HR for highest vs. lowest score quartile = 1.58 [1.10–2.25], p-*trend* = 0.02, Fig. 2B, Table S9).

**Table 5:** Early menarche-derived gene expression signature

Gene symbol	Gene name	Weights
Cell stress response and metabolism		
ATP5B	ATP synthase, H + transporting, mitochondrial F1 complex, beta polypeptide	0.071
ATP5J2	ATP synthase, H + transporting, mitochondrial Fo complex subunit F2	0.141
ATP5J	ATP synthase, H + transporting, mitochondrial Fo complex subunit F6	0.025
BAG1	BCL2 associated athanogene 1	0.025
CHAC1	ChaC glutathione specific gamma-glutamylcyclotransferase 1	− 0.033
DRAM2	DNA damage regulated autophagy modulator 2	0.054
HUS1	HUS1 checkpoint clamp component	0.225
CASQ1	Calsequestrin 1	− 0.066
PTGIS	Prostaglandin I2 synthase	− 0.066
SLC25A4	Solute carrier family 25 member 4	0.108
VDAC2	Voltage dependent anion channel 2	0.064
Cell proliferation, differentiation, and invasion		
GNL3	G protein nucleolar 3	0.005
POP4	POP4 homolog, ribonuclease P/MRP subunit	− 0.384
POLR2E	RNA polymerase II subunit E	0.092
EGFR	Epidermal growth factor receptor	− 0.057
SOX10	SRY-box 10	− 0.065
CENPA	Centromere protein A	0.336
CBX1	Chromobox 1	0.017
FOXO4	Forkhead box O4	− 0.124
GJA5	Gap junction protein alpha 5	− 0.010
IGFBP5	Insulin like growth factor binding protein 5	− 0.039
KRT15	Keratin 15	− 0.024
PDE4DIP	Phosphodiesterase 4D interacting protein	0.043
PHLDB1	Pleckstrin homology like domain family B member 1	− 0.031
TXNL4A	Thioredoxin like 4A	0.021
ZNFX1	Zinc finger NFX1-type containing 1	0.094
Inflammation		
CCL5	C–C motif chemokine ligand 5	0.030
CSF1	Colony stimulating factor 1	− 0.147

## Discussion

In this analysis of women with breast cancer in the NHS and NHSII, early menarche was strongly associated with cancer-promoting molecular changes in both tumor and normal-adjacent tissues, including, most notably, an enrichment of single genes and signaling pathways that drive cell proliferation and are commonly dysregulated in cancer. Similar gene expression differences were corroborated in a subset within TCGA with available data on age at menarche, with several additional pathways related to estrogen response also upregulated. Findings based on PAM50 metrics (molecular subtype, proliferation, risk of recurrence) were consistent with gene expression profiles that suggested association with more aggressive tumor disease. Our 28-gene early menarche-associated gene expression score was suggestively associated with worse survival in the NHS and significantly associated with worse survival in a larger external dataset, METABRIC.

While previous work in the field has focused on understanding the relationship between menarche and breast cancer risk, our gene expression analyses offer insight connecting a classical early life breast cancer risk factor and associations with molecular changes within tumors occurring decades later. We identified 369 individual genes significantly associated with early menarche in tumor-adjacent normal tissues, many of which were associated with cell transformation, invasion, and tumor-promoting changes to the tissue microenvironment. Further investigation into these genes and whether they may hold any biological insights in the tumorigenic process in women that underwent menarche at an early age are warranted. As these normal-adjacent tissues more closely recapitulate the normal breast, it should also be explored whether these genes and the signaling pathways in which they are involved may represent changes that occur early in life. After stratification by ER status, 28 genes in ER-positive tumor tissues were significantly associated with early menarche; these genes ranged in their biological function, with common threads of cell adhesion, DNA damage, and metastatic cell survival. Robust associations linked early menarche with a host of signaling pathways that included proliferation, oxidative stress, cancer cell metabolism, DNA damage repair, and inflammation in both tumor and matched normal tissues in both ER-positive and ER-negative tissues. Of note, the similar pathway enrichment we observed in both ER+ and ER- tissues is concordant with other studies that have shown that early menarche increases risk equally among hormone receptor positive and hormone receptor negative breast cancer subtypes [1, 2, 26–28]*.* As it becomes increasingly apparent that the length of reproductive exposure to estrogens alone may not be the only factor underlying the heightened risk that accompanies early menarche, as was previously believed, more mechanistic work is needed in this area.

In addition to proliferative, metabolic, and stress pathways, we observed associations between early menarche and pathways related to the tissue microenvironment, which included downregulation of myogenesis. Tumor-derived cytokines have been shown to impair myogenesis and alter the skeletal muscle immune microenvironment [29]; indeed, we observed a positive association with pro-inflammatory cytokines and a negative association with myogenesis with early menarche. Normal adjacent tissues also showed an upregulation of epithelial-mesenchymal transition and angiogenesis, both of which promote cancer spread.

Cancer-associated adipocytes are key players in breast cancer progression, undergoing metabolic reprogramming to support tumor cells through secretion of a variety of inflammatory and growth-promoting factors [30]. Early menarche was associated with a significant upregulation of adipogenesis. Women with more adiposity during childhood tend to have earlier menarche, even though early life body fatness has been associated with reduced breast cancer risk [31]. In the NHS, we recently showed that women with higher body fatness during childhood/adolescence was associated with the downregulation of pathways involved in cell stress, proliferation, and inflammation in breast tumors. 11 pathways identified for early life body fatness overlapped with our findings for early menarche, including 6 proliferation-related, 3 cell stress-related, and 2 microenvironment-related pathways. Interestingly, all show distinctly inverse directionality, which is consistent with the known complex relationships between early life body size, menarche, and breast cancer risk.

Though sample size was limited in TCGA, we observed trends and patterns in the tumor signaling pathway analyses similar to the NHS cohort. Four out of 10 of the significant pathways enriched in tumors from the TCGA directly overlapped with those identified within the NHS cohort, including upregulation of numerous cancer cell proliferation-, metabolism-, and inflammation-related pathways. In addition to those that directly replicated, tumors from women in the TCGA study population also exhibited a significant upregulation of distinct but related signaling pathways related to innate immune response, including complement, coagulation, and allograft rejection. Interestingly, early and late estrogen signaling was also upregulated in TCGA tumors from women with early menarche. As previously discussed, some have postulated that the increased breast cancer risk associated with early menarche may relate, in part, to higher levels and a longer exposure window to mitogenic estrogens. This hypothesis aligns with our data showing increased estrogen signaling in tumors was detected from women who underwent early menarche, even when adjusted for hormone receptor status. Some recent evidence also suggests that among women who experience menarche at an early age, those who have single nucleotide polymorphisms within genes involved in estrogen signaling are at higher risk of breast cancer than those who do not [32]. Overall, our findings within TCGA closely mirror our data within the NHS cohort and support the conclusion that early menarche is associated with more proliferative and pro-tumorigenic breast tumor characteristics. While our study focuses on early menarche, which represents exposure to estrogen early in life, other reproductive risk factors can also act to increase estrogen levels throughout the life course (e.g. parity, breast-feeding, exogenous hormone use). Therefore, further investigation to understand whether they may impact tumor biology in a similar or distinct manner would be of interest.

The increased proliferation and other tumor-promoting pathways discovered in our gene expression analyses led us to hypothesize that tumors from women with early menarche may possess a more aggressive tumor phenotype. Leveraging PAM50 subtypes, we found early menarche was associated with basal-like and HER2-enriched intrinsic molecular subtypes, which are considered more aggressive diseases with worsened prognosis [24], higher tumor proliferative index, and increased risk of recurrence score, which has been found to be highly predictive of risk of distant relapse, performing better than other methods of risk prediction [23], together bolstering our suspicions that these tumors may possess more aggressive molecular and pathological features. Concordantly, our NHS-derived gene expression signature that captures the molecular characteristics of tumors from women who experienced early menarche showed a significant, dose–response trend that was suggestively positively associated with cancer recurrence in NHS and significantly associated in METABRIC. This suggests our menarche-derived gene expression signature captured the molecular tumor features and associated prognostic impact in breast cancer patients who experienced early menarche.

In this study, we investigated the molecular features of breast tumors in relation to age at menarche, an established epidemiologic breast cancer risk factor. Early menarche was associated with more aggressive tumor molecular subtypes and characteristics, and our early menarche-derived gene expression signature was associated with a higher risk of breast cancer recurrence. Together, these results highlight how a common and increasingly prevalent early life exposure may influence the molecular and pathological phenotype of breast tumors later in life and how these changes relate to breast cancer prognosis. As the age of menarche onset continues to decline, better understanding of its influence on breast tumor biology and prognosis may lead to novel secondary prevention strategies in the future.

### Supplementary Information

Additional file1 

Additional file2 

Additional file3 

Additional file4 

Additional file5 

Additional file6 

Additional file7 

Additional file8 

Additional file9 

Additional file10 

## Data Availability

Gene expression data analyzed in this study was deposited in Gene Expression Omnibus and is publicly available (GPL22920, GSE93601, GSE115577).

## References

[1] Hamajima N (2012). Menarche, menopause, and breast cancer risk: Individual participant meta-analysis, including 118 964 women with breast cancer from 117 epidemiological studies. Lancet Oncol.

[2] Britt K (2012). Menarche, menopause, and breast cancer risk. Lancet Oncol.

[3] Fuhrman BJ (2021). Association of the age at menarche with site-specific cancer risks in pooled data from nine cohorts. Cancer Res.

[4] Liang J, Shang Y (2013). Estrogen and cancer. Annu Rev Physiol.

[5] McDowell MA, Brody DJ, Hughes JP (2007). Has age at menarche changed? Results from the National Health and Nutrition Examination Survey (NHANES) 1999–2004. J Adolesc Health.

[6] Martinez GM (2020). Trends and patterns in menarche in the United States: 1995 through 2013–2017. Natl Health Stat Report.

[7] Poole EM (2011). Body size in early life and adult levels of insulin-like growth factor 1 and insulin-like growth factor binding protein 3. Am J Epidemiol.

[8] Peng C (2020). Prediagnostic 25-hydroxyvitamin D concentrations in relation to tumor molecular alterations and risk of breast cancer recurrence. Cancer Epidemiol Biomarkers Prev.

[9] Martinez GM. National Health Statistics Reports, Number 146, September 10, 2020. https://www.cdc.gov/nchs/products/index.htm (1995).

[10] Heng YJ (2019). Molecular mechanisms linking high body mass index to breast cancer etiology in post-menopausal breast tumor and tumor-adjacent tissues. Breast Cancer Res Treat.

[11] Kensler KH (2019). PAM50 molecular intrinsic subtypes in the nurses’ health study cohorts. Cancer Epidemiol Biomarkers Prev.

[12] Johnson WE, Li C, Rabinovic A (2007). Adjusting batch effects in microarray expression data using empirical Bayes methods. Biostatistics.

[13] Kensler KH (2019). Androgen receptor expression and breast cancer survival: results from the nurses’ health studies. J Natl Cancer Inst.

[14] Baer HJ (2011). Risk factors for mortality in the nurses’ health study: a competing risks analysis. Am J Epidemiol.

[15] Smyth GK (2004). Linear models and empirical bayes methods for assessing differential expression in microarray experiments. Stat Appl Genet Mol Biol.

[16] Leek JT, Storey JD (2007). Capturing heterogeneity in gene expression studies by surrogate variable analysis. PLoS Genet.

[17] Benjamini Y, Hochberg Y (1995). Controlling the false discovery rate: a practical and powerful approach to multiple testing. J Roy Stat Soc Ser B (Methodol).

[18] Wu D, Smyth GK (2012). Camera: a competitive gene set test accounting for inter-gene correlation. Nucleic Acids Res..

[19] Heng YJ (2020). The association of modifiable breast cancer risk factors and somatic genomic alterations in breast tumors: the cancer genome atlas network. Cancer Epidemiol Biomark Prev.

[20] Wang J (2017). Alcohol consumption and breast tumor gene expression. Breast Cancer Res.

[21] Wallden B (2015). Development and verification of the PAM50-based Prosigna breast cancer gene signature assay. BMC Med Genom.

[22] Nielsen TO (2010). A comparison of PAM50 intrinsic subtyping with immunohistochemistry and clinical prognostic factors in tamoxifen-treated estrogen receptor-positive breast cancer. Diagnosis.

[23] Parker JS (2009). Supervised risk predictor of breast cancer based on intrinsic subtypes. J Clin Oncol.

[24] Kensler KH (2019). PAM50 molecular intrinsic subtypes in the nurses’ health study cohorts. Cancer Epidemiol Biomark Prevent.

[25] Friedman J, Hastie T, Tibshirani R (2010). Regularization paths for generalized linear models via coordinate descent. J Stat Softw.

[26] Hamajima N (2012). Menarche, menopause, and breast cancer risk: Individual participant meta-analysis, including 118 964 women with breast cancer from 117 epidemiological studies. Lancet Oncol.

[27] Jung AY (2022). Distinct reproductive risk profiles for intrinsic-like breast cancer subtypes: pooled analysis of population-based studies. J Natl Cancer Inst.

[28] Ritte R (2013). Height, age at menarche and risk of hormone receptor-positive and -negative breast cancer: a cohort study. Int J Cancer.

[29] Hogan KA (2018). Tumor-derived cytokines impair myogenesis and alter the skeletal muscle immune microenvironment. Cytokine.

[30] Wu Q (2019). Cancer-associated adipocytes: key players in breast cancer progression. J Hematol Oncol.

[31] Baer HJ (2005). Body fatness during childhood and adolescence and incidence of breast cancer in premenopausal women: a prospective cohort study. Breast Cancer Res.

[32] Song SS, Kang S, Park S (2022). Association of estrogen-related polygenetic risk scores with breast cancer and interactions with alcohol intake, early menarche, and nulligravida. Asian Pac J Cancer Prev.

