# Supplementary material for: Association of early menarche with breast tumor molecular features and recurrence
Source: Breast Cancer Res. 2024 Jun 17;26:102. doi: 10.1186/s13058-024-01839-0 (PMC11181557; doi:10.1186/s13058-024-01839-0)
Supplement: Supplementary file 1 — Additional file1 [file 13058_2024_1839_MOESM1_ESM.docx]

**Supplemental Figure 1.** **Study workflow of gene expression analyses**


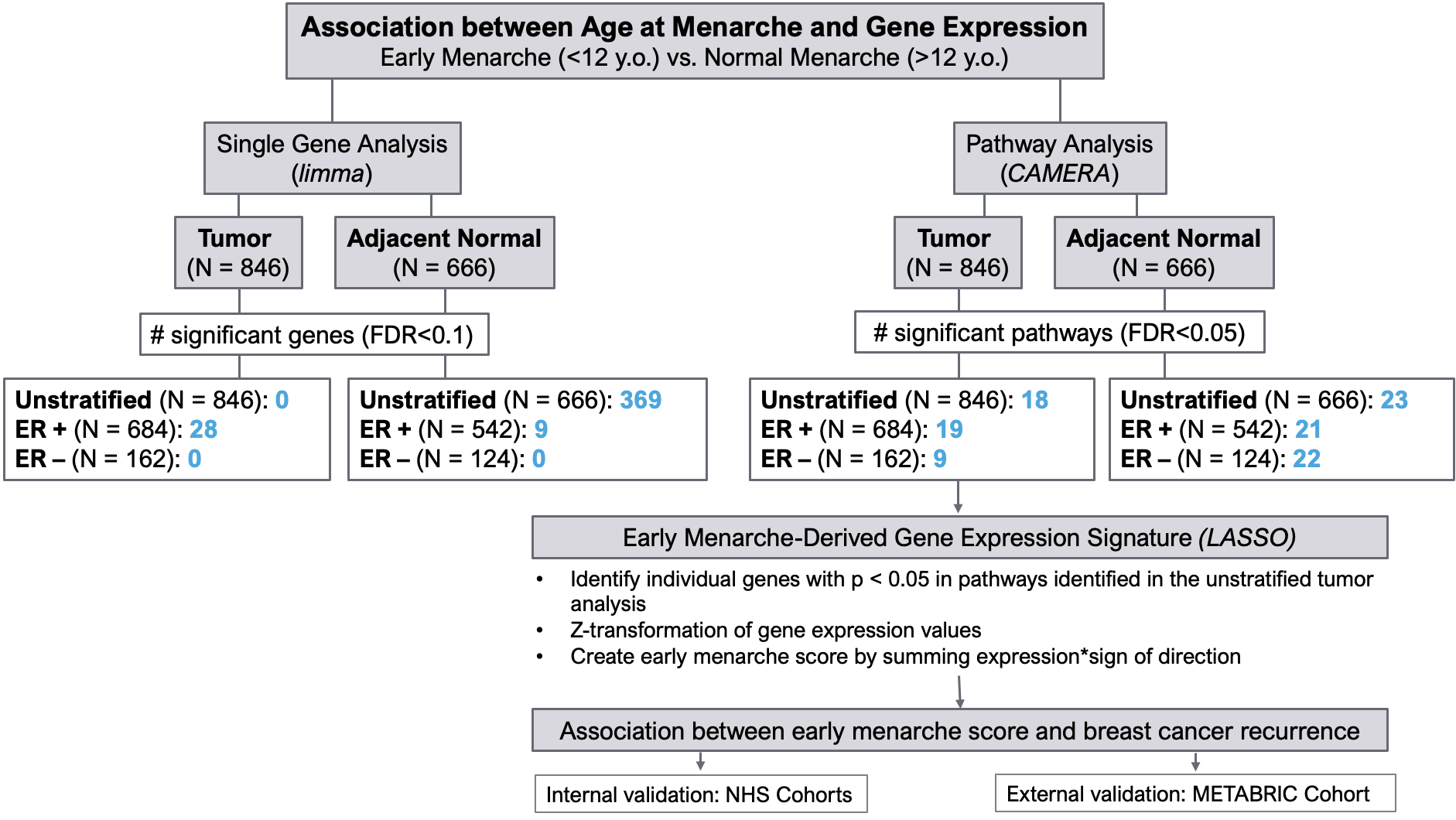


# significant genes (FDR < 0.1)

# significant pathways (FDR < 0.1)
