# Supplementary material for: Association of early menarche with breast tumor molecular features and recurrence
Source: Breast Cancer Res. 2024 Jun 17;26:102. doi: 10.1186/s13058-024-01839-0 (PMC11181557; doi:10.1186/s13058-024-01839-0)
Supplement: Supplementary file 5 — Additional file5 [file 13058_2024_1839_MOESM5_ESM.docx]

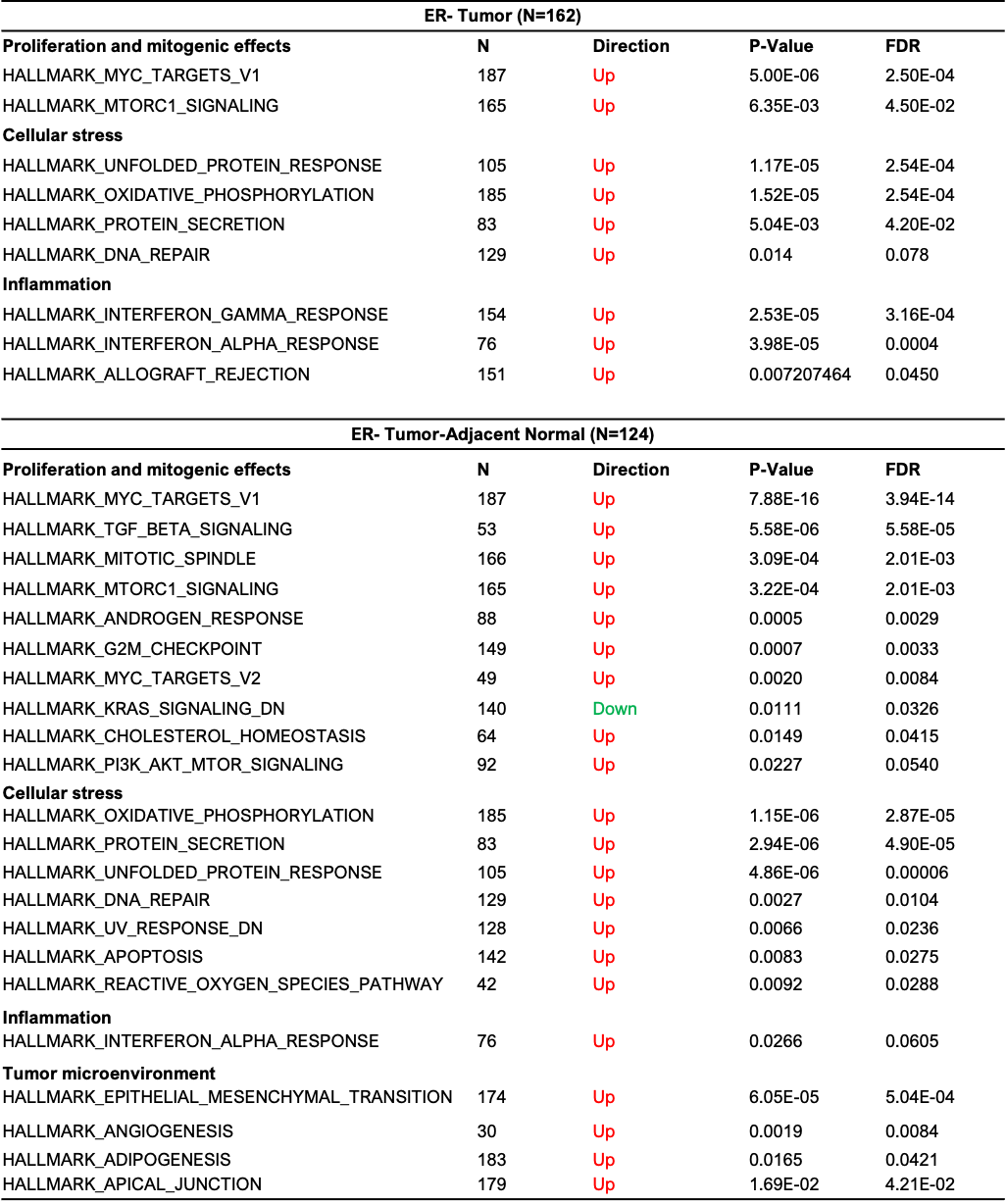


**Note**: Each regression model was adjusted for the following covariates, selected *a priori*: age at breast cancer diagnosis (continuous), year of diagnosis (continuous), tumor stage (1-4), chemotherapy (yes/no/unknown), radiation (yes/no/unknown), endocrine therapy (yes/no/unknown), oral contraceptive use (current-/past-/never-user/unknown), race (white/non-white), parity (continuous), BMI at 18 (continuous), weight change (BMI at diagnosis – BMI at 18), and physical activity at time of diagnosis (continuous). Age at menarche was dichotomized and modeled as a categorical variable of “early” (< 12 years old) vs. “not early” (> 12 years old).

**Table S4 | Pathway enrichment analysis of age at menarche in ER- breast tumors and ER- normal-adjacent tissues.**
